# Cell-free riboswitches

**DOI:** 10.1039/d1cb00138h

**Published:** 2021-08-04

**Authors:** Takeshi Tabuchi, Yohei Yokobayashi

**Affiliations:** Nucleic Acid Chemistry and Engineering Unit, Okinawa Institute of Science and Technology Graduate University Onna Okinawa 904-0495 Japan yohei.yokobayashi@oist.jp

## Abstract

The emerging community of cell-free synthetic biology aspires to build complex biochemical and genetic systems with functions that mimic or even exceed those in living cells. To achieve such functions, cell-free systems must be able to sense and respond to the complex chemical signals within and outside the system. Cell-free riboswitches can detect chemical signals *via* RNA–ligand interaction and respond by regulating protein synthesis in cell-free protein synthesis systems. In this article, we review synthetic cell-free riboswitches that function in both prokaryotic and eukaryotic cell-free systems reported to date to provide a current perspective on the state of cell-free riboswitch technologies and their limitations.

## Abstract 2

Synthetic riboswitches can be used as chemical gene switches in cell-free protein synthesis systems. We provide a current perspective on the state of cell-free riboswitch technologies and their future directions.

## Graphical abstract

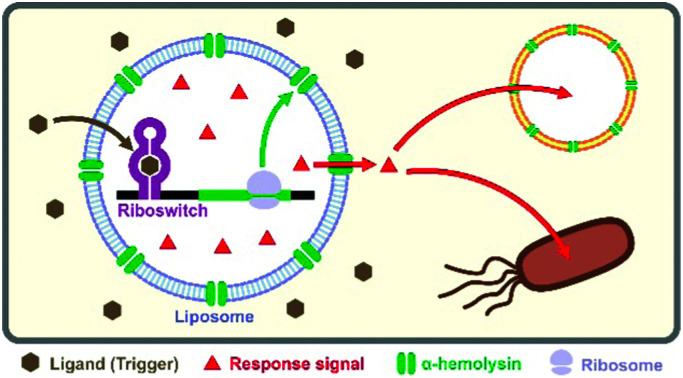

## 1. Introduction

Riboswitches are gene switches composed of ribonucleic acid (RNA). They have been found in diverse species of bacteria and in few eukaryotes, and they are used to sense and respond to a variety of metabolites such as coenzymes, nucleobases, ions, and amino acids.^1–5^ A canonical bacterial riboswitch consists of an aptamer domain and an expression platform that are located in the 5′ untranslated region (UTR) of an mRNA. The aptamer domain directly and specifically binds a small molecule metabolite while the expression platform mediates a local structural change of the mRNA upon aptamer–ligand binding. The structural change results in upregulation or downregulation of expression of the protein(s) encoded in the same mRNA.^1,2,4–10^ It has been suggested that these riboswitches may be remnants of the ancient RNA world.^8,11^

The possibility of chemical regulation of gene expression without direct involvement of proteins (*e.g.*, transcription factors) has inspired many researchers to develop synthetic riboswitches that respond to non-natural molecules using RNA aptamers selected *in vitro*. While many synthetic riboswitches mimicking natural mechanisms have been designed in bacteria, synthetic riboswitches that function in other cells and organisms, and riboswitches based on mechanisms not found in nature have also been developed.^9,12–19^

Bottom-up or cell-free synthetic biology is emerging as a frontier in synthetic biology whose goal is to design and build biochemical or genetic networks (cell-free systems) that display complex functions without using living cells.^20–22^ Building such cell-free systems can improve our understanding of the design principles of biological systems. Alternatively, with fewer experimental constraints compared to living cells, researchers may be able to build cell-free systems with functions or properties that are difficult or impossible using *in vivo* systems, for example, production of toxic secondary metabolites or hybrid systems with nonbiological components. Cell-free protein synthesis (CFPS) systems based on cell extracts or a reconstituted translational apparatus play key roles in these efforts. Synthetic riboswitches that can function in these cell-free platforms are attractive tools for interfacing the cell-free systems with diverse chemical signals.

Cell-free riboswitches, however, have not received as much attention as the synthetic riboswitches that function in living cells. To our knowledge, no comprehensive reviews focusing on cell-free riboswitches have been published. Consequently, we surveyed the literature on synthetic cell-free riboswitches that function in CFPS systems to provide a current perspective on the various types of cell-free riboswitches designed and their emerging applications. The cell-free riboswitches discussed in this review are summarized in Tables 1 and 2.

**Table 1:** List of riboswitches in prokaryotic cell-free systems

Mechanism	Riboswitch	Ligand	ORF	On/off ratio (ligand conc.)	CFPS*	Year	Ref.
Aptazyme-driven RBS release	duplex5-Theo	Theophylline	Luc	ON ∼9 × (2 mM)	PURE^a^	2007	36
			LacZ	ON ∼30 × (2 mM)			
	duplex5-cGMP	cGMP	Luc	ON ∼10 × (10 mM)	PURE^a^	2007	36
	DelC-M3	TPP	GFP	ON ∼48 × (0.3 mM)	PURE^c^	2012	39
	Theo/HHR	Theophylline	GFP	ON ∼15 × (2 mM)	PURE^c^	2012	39
Nonsense suppression	AST4m	Theophylline	Luc	ON 11.6 × (1 mM)	PURE^b^	2008	41 and 115
RBS sequestration	pLac-thiM#2	TPP	GFPuv	ON 6 × (1 mM)	CE^f^	2009	42
			mCherry	ON 14 × (1 mM)			
			Luc	ON 20 × (1 mM)			
	pLac-tenA#59	TPP	Luc	ON 6 × (1 mM)	CE^f^	2009	42
	pT7-theo	Theophylline	YPet	ON 6 × (500 μM)	PURE^d^ + AC^i^^j^	2011	43 and 107
	pTac-theo	Theophylline	YPet	ON 8 × (500 μM)	CE^f^ + AC^i^^j^	2011	43
	JF001A	Theophylline	αHL	ON ∼10 × (1.5 mM)	PURE^d^ + AC^j^	2014	108
	pTac-C	Theophylline	Luc	ON ∼65 × (2 mM)	CE^f^	2014	44
	TMR-10	TMR	Luc	ON ∼16.5 × (30 μM)	CE^f^	2014	46
	Dopa-5	Dopamine	Luc	ON 2 × (1 mM)	CE^f^	2014	46
	T4-2	Thyroxine	Luc	ON 2.4 × (150 μM)	CE^f^	2014	46
	Theo-αHL	Theophylline	αHL	N/D	CE^g^ + AC^j^	2017	109
	H2	Histamine	mCherry	ON 30.7 × (5 mM)	PURE^e^ + AC^j^	2019	45
			αHL	N/D			
			PLC	N/D			
	Theo-GFP-MG	Theophylline	sfGFP	ON ∼12 × (10 mM)	CE^h^, PURE^d^	2021	31
Transcription termination	pTac-ade/ydhL	Adenine	YPet	ON 1.7 × (1 μM)	CE^f^ + AC^i^^j^	2011	43
	FRR/CrcB	Fluoride (F^−^)	sfGFP	ON ∼20 × (3.5 mM)	CE^g^	2020	48
			C23DO	N/D			

a PURESYSTEM classic II (Post-Genome Institute).

b PURESYSTEM custom (Post-Genome Institute) without RF1.

c Modified Shimizu's PURE system with T7 RNAP replaced by T3 RNAP.

d PURExpress (New England Biolabs).

e PURE*frex* 1.0 (Gene Frontier). CE: cell extracts.

f Commercial S30 cell extract from *E. coli* B strain SL119 (Promega).

g In-house prepared *E. coli* cell extract from Rosetta2 (DE3).

h In-house prepared *E. coli* cell extract from BL21 Star (DE3). AC: artificial cells.

i Water-in-oil emulsions.

j Lipid vesicles/liposomes. cGMP: cyclic guanosine monophosphate, TMR: tetramethylrhodamine, TPP: thiamine pyrophosphate, αHL: α-hemolysin, C23DO: catechol-2,3-dioxygenase, GFP: green fluorescent protein (sf-: super-folder, -uv: ultraviolet), LacZ: β-galactosidase, Luc: firefly luciferase, PLC: phospholipase C, RNAP: RNA polymerase, YPet: yellow fluorescent protein for energy transfer.

**Table 2:** List of riboswitches in eukaryotic cell-free systems

Mechanism	Riboswitch	Ligand	ORF	On/off ratio (ligand conc.)	CFPS*	Year	Ref.
Ribosome blocking	tob3-RSETA	Tobramycin	RSETA	OFF ∼7 × (60 μM)	WGE^a^	1998	76
	H2-RSETA	H33258	RSETA	OFF ∼12 × (80 μM)	WGE^a^	1998	76
	(Th)_3_	Theophylline	CAT	OFF ∼8 × (1 mM)	WGE^a^	2002	77
	B_3_	Biotin	CAT	OFF ∼9 × (1 mM)	WGE^a^, RRL	2002	77
	th+0-U7	Theophylline	YPet	OFF 5.9 × (1 mM)	WGE^b^	2018	78
			NLuc	OFF 7.2 × (1 mM)			
	TMR+0-U7	TMR	NLuc	OFF 5.1 × (500 μM)	WGE^b^	2018	78
Aptazyme-regulated translation	mRNA6-Theo	Theophylline	Luc	ON ∼50 × (500 μM)	WGE^b^	2009	87
	mRNA6-cGMP	cGMP	Luc	ON ∼10 × (750 μM)	WGE^b^	2009	87
IRES-modulated translation	theo5	Theophylline	Luc	ON 9.6 × (1 mM)	WGE^b^	2011	88 and 116
			eGFP	ON 5.1 × (1 mM)			
	FMN4	FMN	Luc	ON 7.5 × (300 μM)	WGE^b^	2011	88
	tc7	Tetracycline	Luc	ON 29 × (300 μM)	WGE^b^	2011	88
			eGFP	ON 35 × (300 μM)			
	sr4	SRB	Luc	ON 4.2 × (300 μM)	WGE^b^	2011	88
	theoN5	Theophylline	NLuc	OFF 5.8 × (1 mM)	WGE^b^	2012	89
	tc-N5	Tetracycline	NLuc	OFF 4.9 × (300 μM)	WGE^b^	2012	89
	FMN-N5	FMN	NLuc	OFF 4.7 × (300 μM)	WGE^b^	2012	89
	theoA3-rS	Theophylline	Luc	OFF 14 × (1 mM)	WGE^b^	2017	90
	nDNA-547-N4	nDNA	NLuc	ON 21 × (300 μM)	WGE^b^	2020	114
Ribosomal shunting	theoS1	Theophylline	Luc	ON 14.4 × (1 mM)	WGE^b^	2013	96 and 117
	tmrS1	TMR	Luc	ON 5.4 × (333 μM)	WGE^b^	2013	96
Nonsense suppression	theo(th1)-MS(4)	Theophylline	Luc	ON 7.8 × (1 mM)	WGE^b^	2015	97 and 115
	tc(th1)-MS(4)	Tetracycline	Luc	ON 81 × (100 μM)	WGE^b^	2015	97 and 115
	FMN(th1)-MS(4)	FMN	Luc	ON ∼4 × (30 μM)	WGE^b^	2015	97 and 115
3′ CITE-modulated translation	5SL-BYm2-theo	Theophylline	YPet	ON 7.7 × (1 mM)	WGE^b^	2017	99
			NLuc	ON 7.3 × (1 mM)			
	5SL-BYm2-TMR	TMR	NLuc	ON 5.8 × (100 μM)	WGE^b^	2017	99

a Promega.

b WEPRO1240 (CellFree Sciences). RRL: rabbit reticulocyte lysate system (Promega). cGMP: cyclic guanosine monophosphate, FMN: flavin mononucleotide, H33258: Hoechst dye 33258, nDNA: nano-sized ssDNA or pentadeoxyribonucleotide, TMR: tetramethylrhodamine, SRB: sulphorhodamine B, CAT: chloramphenicol acetyltransferase, eGFP: enhanced green fluorescent protein, Luc: firefly luciferase, NLuc: NanoLuc, RSETA: ORF of the undigested cloning site from pRSET-A plasmid (Invitrogen), YPet: yellow fluorescent protein for energy transfer.

## 2. Cell-free systems for riboswitch research

Synthetic riboswitches have been designed and studied in both prokaryotic and eukaryotic CFPS systems. Prokaryotic CFPS systems that have been used for cell-free riboswitches include conventional *Escherichia coli* lysate-based systems (S30 extracts),^23–26^ and reconstituted *in vitro* translation systems based on the PURE (stands for Protein synthesis Using Recombinant Elements) system originally developed by Shimizu *et al.*^27,28^ S30 extracts are relatively inexpensive and simple to produce, can be scaled up, and exhibit high protein yields.^26,29,30^ The extracts also contain numerous nonessential cellular components and may reflect the intracellular environment more accurately. PURE systems, on the other hand, consist of purified, mostly recombinant, components from *E. coli*, therefore, are much more expensive and less scalable. However, they offer a flexible, well-defined, and reproducible platform that allows more freedom to control biochemical parameters if desired. The reconstituted systems also contain low amounts of ribonucleases which can significantly influence riboswitch performance.^31^

Although a number of eukaryotic CFPS systems are currently available, nearly all of the eukaryotic cell-free riboswitches have been studied using wheat germ extract (WGE). In dry state, wheat germ embryos naturally contain all the components required for translation, ready to start protein synthesis as germination begins.^32,33^ The Endo group developed a stable WGE system with high translation efficiency after removing endogenous translation inhibitory components that limited the life span of the conventional WGEs.^34^ The current WGE systems offer the highest translation efficiency among eukaryotic CFPS systems^35^ and can produce high quality proteins in folded state.^32^ The complexity of translation regulation mechanisms in eukaryotes compared to those in prokaryotes presents both unique challenges and opportunities in designing eukaryotic cell-free riboswitches.

## 3. Prokaryotic cell-free riboswitches

### 3.1. Aptazyme-based riboswitches

**Fig. 1:**
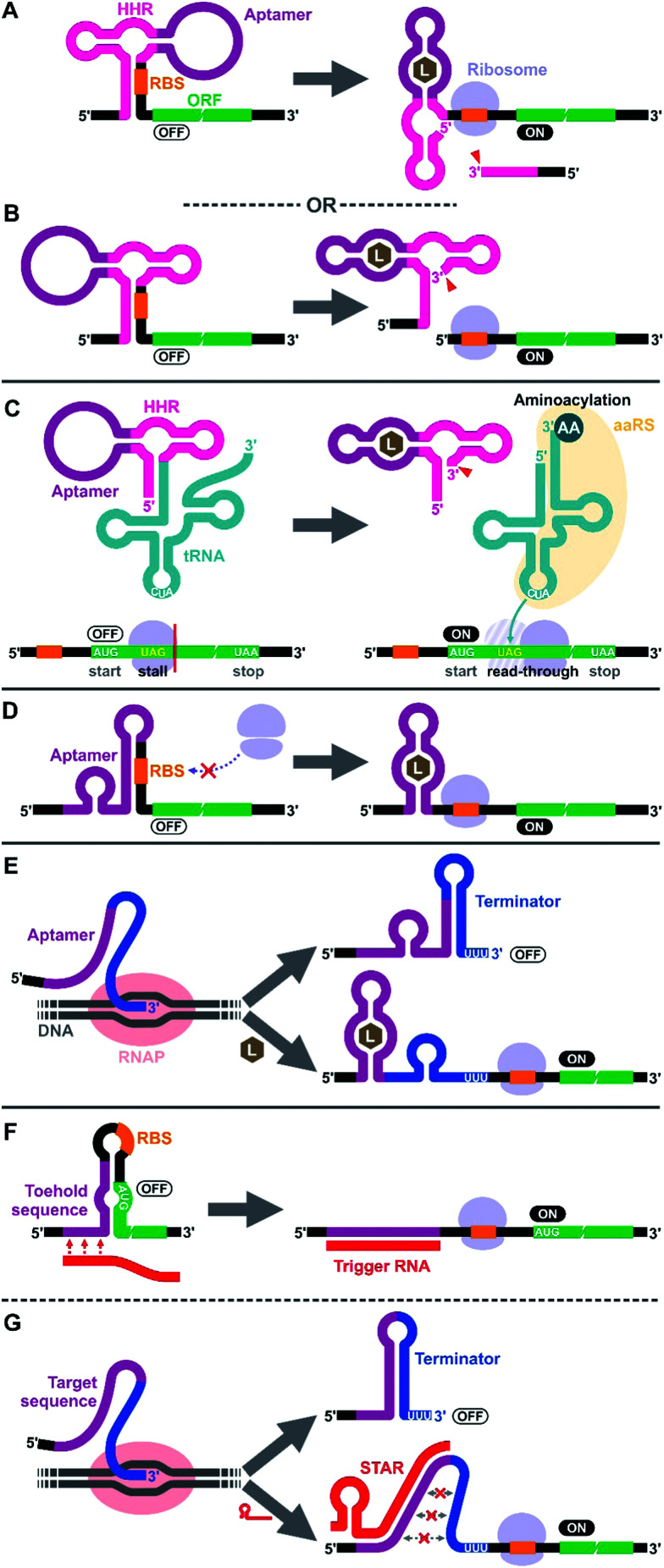
Riboswitch mechanisms in prokaryotic cell-free systems. (A and B) Aptazyme-driven RBS release: aptamer–ligand binding activates self-cleavage of the ribozyme resulting in physical separation of the anti-RBS sequence from the mRNA. (C) Aptazyme-driven sup-tRNA release: self-cleavage of the ribozyme upon aptamer–ligand binding releases the sup-tRNA which undergoes aminoacylation and allows translation elongation past the amber stop codon (UAG). (D) RBS sequestration: aptamer–ligand binding induces a structural change near the RBS modulating the accessibility of the sequence to ribosomes. (E) Regulation of transcription termination: co-transcriptional folding of the riboswitch in the presence of the ligand prevents formation of the transcription terminator hairpin structure, allowing the full-length mRNA to be transcribed and the protein to be translated. (F and G) DNA/RNA-responsive switches: they employ similar regulatory mechanisms as riboswitches but respond to specific DNA or RNA sequences through Watson–Crick base pairing. Toehold switches regulate translation initiation (F), while small transcription activating RNAs (STARs) regulate transcription termination (G). AA: amino acid, aaRS: aminoacyl tRNA synthetase, HHR: hammerhead ribozyme, L: ligand, RBS: ribosome binding site, RNAP: RNA polymerase.

To our knowledge, the first synthetic cell-free riboswitch in a prokaryotic CFPS system was reported by Ogawa and Maeda in 2007 using the PURE system.^36^ In this work, the researchers adapted the theophylline-activated hammerhead ribozyme (aptazyme) reported by the Breaker group^37,38^ so that the ribosome binding site (RBS) of the mRNA was sequestered by a complementary sequence upstream of the ribozyme (anti-RBS). Self-cleavage of the ribozyme in the presence of theophylline releases the RBS, allowing protein translation to be activated (Fig. 1A). They also constructed a riboswitch that responds to cyclic guanosine monophosphate (cGMP) based on the same strategy.^36^ The same theophylline riboswitch and another riboswitch based on a thiamine pyrophosphate (TPP)-activated aptazyme^12,13^ (Fig. 1B) were further analyzed by Kobori *et al.* in a modified PURE system for the purpose of optimizing riboswitch performance based on kinetic modeling.^39^ It should also be noted that synthetic riboswitches developed based on the same design strategy have been shown to function in *E. coli* by Ogawa and Maeda,^40^ and the Hartig group.^12,13^ In another study, Ogawa and Maeda tethered the theophylline-responsive aptazyme to the 5′ end of a nonsense-suppressor tRNA (sup-tRNA) to regulate translation read-through of a gene that contains an amber stop codon in a customized PURE system (Fig. 1C).^41^ In this strategy, the aptazyme activity mimics the canonical 5′ terminus cleavage mediated by RNase P *in vivo* which is absent in the PURE system.

### 3.2. Translationally regulated riboswitches

The majority of prokaryotic cell-free riboswitches operate by regulating the translation efficiency of the associated mRNA in response to aptamer–ligand binding. While the aptazyme-based riboswitches achieve translational regulation by irreversible self-cleavage that physically separates the RBS from the anti-RBS sequence, a class of natural riboswitches regulate the local structure near the RBS through a structural change triggered by aptamer–ligand interaction (Fig. 1D). A series of cell-free riboswitches have been designed inspired by these natural prokaryotic riboswitches (Table 1). Muranaka *et al.* used an *E. coli* S30 extract system to characterize several TPP-responsive synthetic riboswitches that were originally engineered in *E. coli*, and observed comparable riboswitch performance in the cell-free system.^42^ Similarly, a number of cell-free riboswitches of this type were originally engineered in *E. coli* and simply adapted to or further optimized in cell-free systems.^43,44^ In contrast, some cell-free riboswitches have been directly engineered in prokaryotic cell-free systems.^45,46^

### 3.3. Transcriptionally regulated riboswitches

Another major class of natural riboswitches regulate premature transcription termination upstream of the start codon by modulating the transcription terminator structure upon aptamer–ligand binding (Fig. 1E). Although transcriptionally regulated riboswitches represent the most common riboswitch class in bacteria,^47^ there are only two reported cell-free riboswitches of this type, both based on naturally occurring sequences. Martini and Mansy studied the adenine-responsive riboswitch associated with the *ydhL* gene of *Bacillus subtillis* using an *E. coli* S30 extract, and observed modest activation (1.7-fold) of gene expression in the presence of adenine.^43^ More recently, Thavarajah *et al.* used the fluoride-responsive riboswitch that activates expression of the efflux pump CrcB in *B. cereus* in lyophilized *E. coli* cell-free extract in their effort to develop a biosensor for fluoride detection in water.^48^ Both systems used *E. coli* RNA polymerase rather than T7 RNA polymerase for cell-free transcription which is reasonable considering the low termination efficiency of T7 RNA polymerase at canonical bacterial transcription terminators.^49,50^ The scarcity of engineered riboswitches that are transcriptionally regulated also extends to *E. coli* and other bacteria, with only a handful of such riboswitches reported.^51–56^ This could partly be due to the fact that Gram-negative bacteria, including *E. coli*, seem to prefer translationally regulated riboswitches, whereas Gram-positive bacteria favor transcriptionally regulated ones.^5,7,57,58^ Genes of the Gram-positive bacteria are more frequently organized in larger polycistronic operons which are regulated more efficiently by transcriptionally regulated riboswitches.^7,57^

### 3.4. DNA/RNA-responsive cell-free switches

We would like to briefly point out the existence of related cell-free switches/sensors that sequence-specifically respond to nucleic acids (DNA/RNA) in cell-free systems. These switches modulate transcription or translation through mechanisms similar to those of the natural bacterial riboswitches except that they respond to specific DNA or RNA sequences through Watson–Crick base pairing. Earlier examples come from Aoyama and coworkers who employed a molecular beacon like structure to regulate cell-free translation in response to short oligo DNA or RNA.^59,60^ More recent and sophisticated sensors called toehold switches^61^ also regulate cell-free translation in response to specific RNA sequences (Fig. 1F). Small transcription activating RNAs (STARs)^62^ exploit RNA–RNA hybridization to regulate premature transcription termination in cell-free systems (Fig. 1G). These RNA/DNA-responsive cell-free switches have been used to prototype genetic circuits^63–66^ and to develop biosensors for detecting RNAs from viruses,^67,68^ bacteria,^69^ and a variety of RNA markers.^69,70^

## 4. Eukaryotic cell-free riboswitches

### 4.1. Ribosome blocking

Apart from the TPP riboswitches that modulate pre-mRNA splicing in fungi and plants,^6,71–73^ there are no other known natural riboswitches in eukaryotes whose posttranscriptional regulatory mechanisms are very different from those in prokaryotes. Consequently, synthetic cell-free riboswitches that function in eukaryotic CFPS systems have been designed to operate *via* non-natural regulatory mechanisms.

**Fig. 2:**
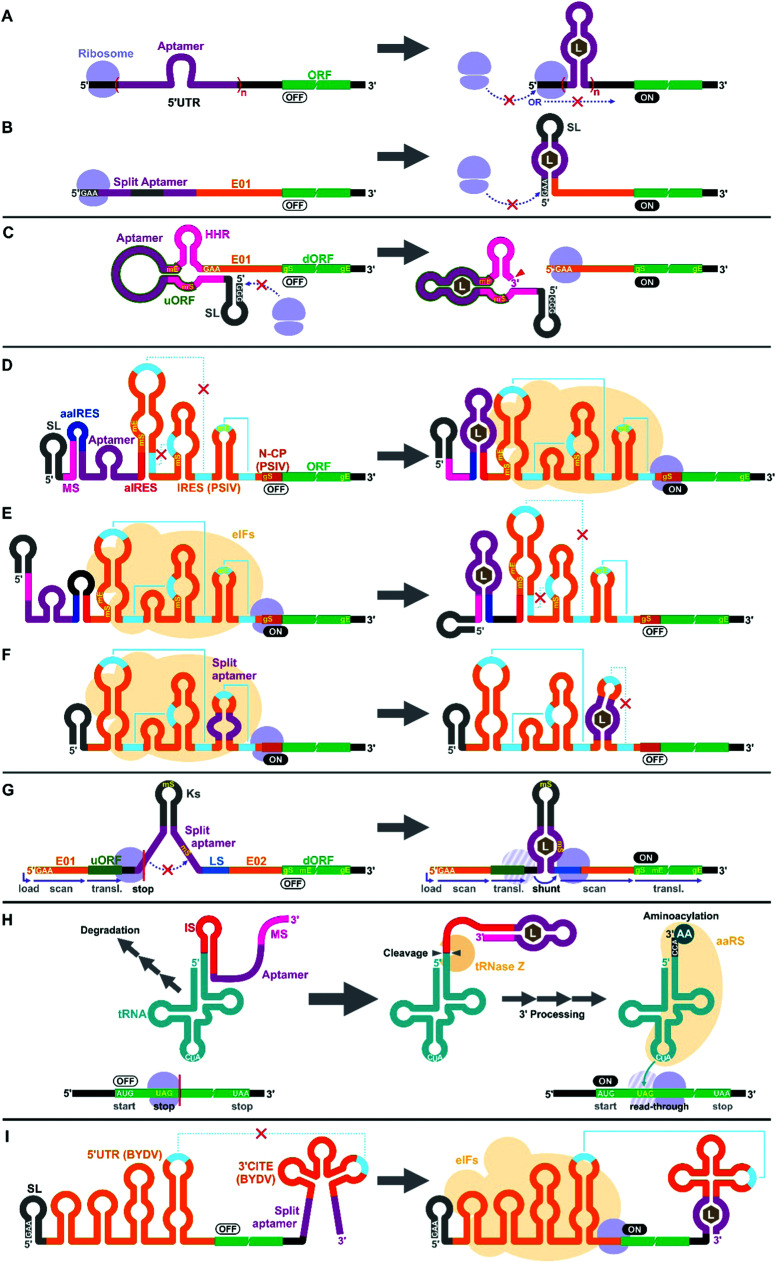
Riboswitch mechanisms in eukaryotic cell-free wheat germ extract (WGE). (A and B) Ribosome blocking: Stable structure induced by aptamer–ligand binding at the 5′ UTR can block ribosome loading or mRNA scanning. (C) Aptazyme-regulated translation: activation of the aptazyme cleaves the mRNA and exposes the 5′ terminus which can be recognized by the WGE's translational machinery. (D–F) IRES-modulated translation: IRES-mediated translation can be controlled by different combinations of sequence elements that include modulator sequence (MS), (split) aptamer, anti-IRES (aIRES), and anti-aIRES (aaIRES). (G) Ribosomal shunting: aptamer–ligand binding brings the upstream ORF (uORF) and the downstream ORF (dORF) to close proximity, allowing the ribosome to shunt over to reinitiate translation from the dORF. (H) Aptamer-regulated sup-tRNA processing: aptamer–ligand binding activates the tRNase Z-mediated 3′ processing of the sup-tRNA–aptamer fusion that would otherwise be rapidly degraded. The mature aminoacylated tRNA suppresses the amber stop codon (UAG) in the coding sequence. (I) 3′ CITE-modulated translation: binding of the ligand to the aptamer promotes correct folding of the 3′ CITE structure to restore the interaction between the 5′ UTR and the 3′ UTR. AA: amino acid, aaRS: aminoacyl tRNA synthetase, BYDV: barley yellow dwarf virus, E01/E02: translational enhancers, eIFs: eukaryotic translation initiation factors, gE: real gene stop codon, gS: real gene start codon, HHR: hammerhead ribozyme, IRES: internal ribosome entry site, IS: inhibitory sequence, Ks: artificial Kozak stem-loop, L: ligand, LS: landing site, mE: mimic stop codon, mS: mimic start codon, N-CP: N-terminus of capsid protein gene, PSIV: *Plautia stali* intestine virus, SL: stem-loop.

Interestingly, earlier reports on eukaryotic cell-free riboswitches predated the discovery of bacterial riboswitches. Inspired by the observation that a stable RNA structure at the 5′ terminus of a eukaryotic mRNA can interfere with ribosome loading or mRNA scanning resulting in lower gene expression,^74,75^ Werstuck and Green inserted an aptamer that binds tobramycin or Hoechst dye 33258 in the 5′ UTR of an mRNA. Addition of the aptamer ligand suppressed translation in WGE presumably due to the stabilization of the aptamer structure upon ligand binding.^76^ Similarly, Harvey *et al.* controlled the synthesis of chloramphenicol acetyltransferase (CAT) in WGE and rabbit reticulocyte lysate by inserting multiple copies of theophylline or biotin aptamers in the 5′ UTR (Fig. 2A).^77^ More recently, Ogawa *et al.* designed split aptamers that induce mRNA stabilization at the 5′ UTR to block ribosome loading in WGE (Fig. 2B).^78^ It is worth noting that similar riboswitch designs have been shown to function in yeast^79–82^ and in mammalian cells.^76^

### 4.2. Aptazyme-based riboswitches

Aptazymes are frequently used to regulate gene expression living eukaryotic cells.^18,83,84^ Ogawa, who reported the first prokaryotic cell-free riboswitches based on an aptazyme also designed the first aptazyme-based cell-free riboswitches in a eukaryotic cell-free system. Incidentally, Ogawa and coworkers have designed the majority of the synthetic riboswitches that function in eukaryotic cell-free systems, specifically, WGE (Table 2). To engineer their aptazyme-based riboswitches, Ogawa exploited a property of the WGE described by Endo and coworkers.^85,86^ WGE can efficiently initiate translation from an uncapped mRNA that contains 5′ GAA trinucleotide followed by an enhancer sequence (E01). Ogawa inserted a theophylline-activated aptazyme directly upstream of the GAA trinucleotide such that the 5′ GAA is exposed upon ribozyme cleavage. Without cleavage, the lack of 5′ GAA, the presence of the relatively stable aptazyme structure, and the presence of a short upstream open reading frame (uORF) embedded within the aptazyme sequence repress reporter gene expression (Fig. 2C).^87^

### 4.3. IRES

Next, Ogawa and coworkers shifted their attention to internal ribosomal entry sites (IRES). An IRES is a viral RNA element that recruits the translation initiation complex in the absence of the 5′ cap structure. Specifically, Ogawa identified the minimum functional sequence of the IRES from *Plautia stali* intestine virus (PSIV), and rationally fused an RNA aptamer to disrupt or induce critical IRES structural elements in the presence of the ligand. For example, Ogawa engineered an ON-switch that activates gene expression in the presence of theophylline following a rational design strategy. First, an 8-nt anti-IRES (aIRES) sequence was introduced upstream of the IRES to disrupt a critical pseudoknot structure, successfully repressing gene expression (OFF). Then, an anti-anti-IRES (aaIRES) and the theophylline aptamer was added further upstream to restore gene expression (ON) by sequestering the aIRES. Finally, a modulator sequence (MS) was appended to the 5′ end of the mRNA interfering with the aaIRES and the aptamer to suppress gene expression (OFF) (Fig. 2D). Ogawa systematically optimized the stability of the MS-aaIRES/aptamer interaction to engineer riboswitches that shift the IRES from the OFF to the ON structure in the presence of theophylline and other ligands.^88^ Ogawa later designed OFF-switches by rearranging the riboswitch elements (Fig. 2E)^89^ demonstrating the flexibility of this design strategy. More recently, Ogawa *et al.* reported a simpler OFF-switch design in which an aptamer was embedded within the IRES structure (Fig. 2F).^90^

### 4.4. Other mechanisms

Ogawa has developed additional cell-free riboswitches in WGE based on other regulatory mechanisms. He exploited the ribosome shunting mechanism used by some viruses to bypass mRNA scanning^91–95^ by incorporating two halves of a split aptamer separated by an intervening sequence. Aptamer–ligand binding brings the two regions to close proximity to induce ribosome shunting, resulting in efficient gene expression (Fig. 2G).^96^

In another design, Ogawa and Tabuchi engineered riboswitches that control readthrough of premature translation termination codons (nonsense amber mutations) using sup-tRNAs in WGE^97^ through a mechanism reminiscent of their aptazyme-based riboswitch that operates in a prokaryotic cell-free system (Fig. 1C).^41^ In the eukaryotic riboswitch, the aptamer was fused to the 3′ end of the sup-tRNA *via* an inhibitory sequence (IS) that interferes with the natural 3′ processing of the tRNA (cleavage by tRNase Z and addition of CCA by tRNA nucleotidyltransferases). Aptamer–ligand interaction exposes the 3′ terminus of the tRNA allowing its rapid maturation followed by suppression of the amber codon (Fig. 2H).^97^ The same design was later adapted by Ogawa *et al.* to develop sup-tRNA switches triggered by complementary DNA oligonucleotides in WGE.^98^

More recently, Ogawa and colleagues manipulated a viral translation initiation mechanism mediated by the 3′ cap-independent translation element (3′ CITE) from barley yellow dwarf virus (BYDV). The viral RNA element located in the 3′ UTR activates translation by forming a kissing loop interaction with another element located in the 5′ UTR, mimicking the canonical 5′ cap-3′ poly(A) interaction mediated by eukaryotic translation initiation factors (eIFs). Ogawa and colleagues inserted a split aptamer within the 3′ CITE to enhance the kissing loop interaction upon aptamer–ligand binding (Fig. 2I).^99^

## 5. Applications of cell-free riboswitches

### 5.1. Biosensors

Cell-free riboswitches possess several potential advantages as a versatile platform for biosensing. First, natural and laboratory evolved RNA aptamers have been reported for a plethora of compounds. Therefore, in principle, one can expect to be able to develop riboswitches for a variety of analytes. While such laboratory evolved aptamers can potentially work in cellular systems, very few aptamers have been used to engineer riboswitches *in vivo*. Various biological factors such as intracellular environment, or cell permeability, stability, or toxicity of the ligand can hinder the development of cellular riboswitches using these aptamers, while cell-free riboswitches can circumvent some of these constraints.

Second, riboswitches can trigger expression of any gene(s), broadening the options for the detection method. Additionally, signal amplification inherent to translation (multiple proteins translated per mRNA molecule) and provided by some reporter genes (enzymes such as luciferase or β-galactosidase) can improve sensitivity of the biosensor. There are some challenges, however, including low stability of CFPS systems and sensitivity to contaminants.

Performance indicators as biosensors such as detection limit, dose–response, and signal-to-noise ratio of some cell-free riboswitches have been analyzed.^36,41,42,46,88,97,100^ However, most of those cell-free riboswitches have never been put to test by measuring more practically relevant samples. A recent notable exception was reported by Thavarajah *et al.* who engineered a cell-free fluoride riboswitch for field application as a biosensor.^48^ They implemented the riboswitch in a lyophilized *E. coli* extract format previously optimized for toehold switches^63,67–69,101^ and used catechol 2,3-dioxygenase (C23DO) as the reporter gene that provides a visual colorimetric output suitable for field use. The practical potential as a biosensor was demonstrated by on-site detection of fluoride levels as low as 50 μM (∼1 ppm) in real-world water samples.^48^

### 5.2. Artificial cells

Artificial cells are emerging as a new frontier in synthetic biology.^102–104^ In many of these artificial cells a CFPS system is encapsulated in a cell-sized compartment along with DNA encoding various genes.^105,106^ Naturally, researchers started building artificial cells that respond to chemical signals by controlling gene expression. Along with canonical transcription factor-based switches well established in bacterial systems (*e.g.*, LacI–IPTG, AraC–arabinose, LuxR–AHL), some synthetic riboswitches have also been introduced in different types of artificial cells such as water-in-oil emulsions,^43,107^ and lipid vesicles.^43,45,107–109^ In a recent report, a riboswitch-regulated DNA was trapped inside silk fibroin-based microcapsules to demonstrate protein expression in the presence of theophylline. However, the riboswitch function was not demonstrated in the protein compartment.^100^

**Fig. 3:**
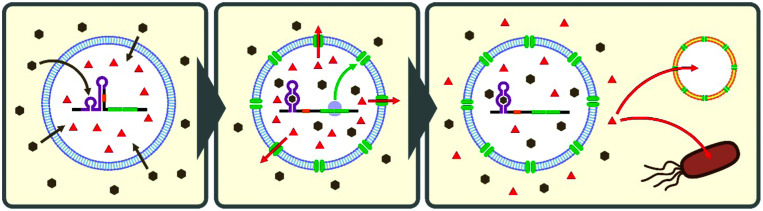
Schematic illustration of artificial cells (*e.g.*, liposomes encapsulating CFPS system) equipped with a cell-free riboswitch. A membrane-permeable ligand (brown hexagons) diffuses into the artificial cells and activates the riboswitch to express α-hemolysin (green ovals). The nanopores localize on the artificial cell membrane allowing non-membrane-permeable small molecules (red triangles) entrapped in the artificial cells to be released. The released compound can serve as a chemical signal to trigger response in other artificial cells or living cells.

Theophylline riboswitches have been used in lipid vesicle-based artificial cells to mediate chemical signaling between artificial cells and bacteria. In this work, Lentini *et al.* encapsulated IPTG inside liposomes along with DNA encoding an α-hemolysin gene controlled by a theophylline-activated riboswitch.^108^ Addition of theophylline triggers expression of α-hemolysin which forms nanometer sized pores on the membrane of the artificial cells. The pores allow IPTG to diffuse out of the artificial cells and to induce GFP expression in co-cultured *E. coli* cells. Similarly, Adamala *et al.* constructed riboswitch-controlled artificial cells that release doxycycline to achieve communication between different artificial cells (Fig. 3).^109^ However, in both examples, performance of the theophylline-responsive riboswitches was not directly characterized in the artificial cells.

More recently, Dwidar *et al.* applied Systematic Evolution of Ligands by EXponential enrichment (SELEX)^110,111^ to discover a novel aptamer that recognizes histamine, and they designed and optimized histamine-responsive cell-free riboswitches in the PURE system.^45^ They thoroughly characterized the riboswitch in liposome-based artificial cells using fluorescence microscopy and flow cytometry to confirm the robust and dynamic response to histamine. Finally, artificial cells that express α-hemolysin or phospholipase C under the control of their riboswitch were constructed to demonstrate either molecular cargo release or self-destruction triggered by histamine.

### 5.3. Mechanistic understanding of riboswitches

Building and analyzing cell-free riboswitches can lead to fundamental insights into the regulatory mechanisms and the design principles of natural and synthetic riboswitches. An advantage of working with cell-free riboswitches is the presence of fewer experimental constraints compared to working with riboswitches in living cells. For example, researchers can precisely control various parameters such as ligand concentration and the timing of ligand addition. Cell-free riboswitches can also circumvent other biological constraints such as ligand toxicity, ligand permeability, and metabolic burden.

**Fig. 4:**
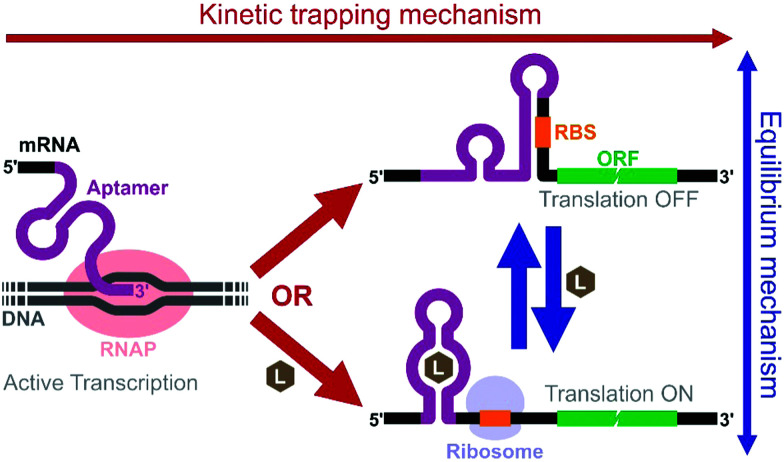
Mechanistic models of riboswitches. In the kinetic trapping mechanism, presence of the ligand during transcription is necessary for the aptamer/riboswitch structure to form. In the thermodynamic equilibrium mechanism, the active (on) and inactive (off) riboswitch structures are in dynamic equilibrium. Therefore, addition of the ligand can shift the equilibrium and affect protein expression even after transcription. L: ligand, RBS: ribosome binding site, RNAP: RNA polymerase.

An elegant example by Mishler and Gallivan illustrates the advantage of using a cell-free system to study riboswitch mechanisms.^44^ The researchers used an *E. coli* S30 extract to study their theophylline-activated riboswitches that they previously engineered in bacteria. By adding theophylline during or after transcription, they unambiguously showed that theophylline must be available during transcription for their riboswitches to function, demonstrating that the kinetic trapping mechanism plays a more dominant role than the thermodynamic equilibrium mechanism for the activation of their riboswitch (Fig. 4). They also showed that the high concentration of theophylline needed to activate the riboswitches was due to this kinetic trapping mechanism and not due to the low permeability of theophylline through the cell membrane as was previously suggested.^112,113^

Independent reports from Kobori *et al.*^39^ and Chushak *et al.*^31^ illustrate how kinetic modeling of riboswitch mechanisms based on experimental data of cell-free riboswitches can provide not only insights into the riboswitch mechanisms, but also hints for optimizing riboswitch performance. Espah Borujeni *et al.* built a more detailed model considering additional kinetic and biophysical parameters such as co-transcriptional folding and free energy of ribosome binding.^46^ Importantly, they used the model to automate riboswitch design using arbitrary aptamers, and they experimentally validated the designed riboswitches in either *E. coli* cells or cell-free S30 extracts. While the designed cell-free riboswitches showed mixed performance, the ability of such a model-driven design to generate functional riboswitches is an important achievement.

## 6. Conclusions and future directions

As reviewed in this article, while a steady number of cell-free riboswitches have been reported over the last two decades, cell-free riboswitches have attracted relatively little attention compared to cellular riboswitches. This trend may change in the near future with the growing interest in cell-free synthetic biology and its applications. With fewer experimental constraints compared to living cells, cell-free systems can allow more precise and extensive control over the genetic circuits and metabolic pathways built by the researchers. Such cell-free systems, much like their cell-based counterparts, will need gene switches to sense and respond to a variety of chemical signals. As discussed above, riboswitches have a number of favorable characteristics as cell-free gene switches.

It should be acknowledged, however, that cell-free riboswitches still need more efforts to prove that they are valuable tools for cell-free synthetic biology. For example, OFF-switches are conspicuously missing in the prokaryotic cell-free systems (Table 1). While several cell-free riboswitches show high ON/OFF ratios greater than 30, many others display more modest responses. Nonetheless, the variety of ligands that cell-free riboswitches have been engineered to detect are quite broad compared to cell-based riboswitches. Although theophylline has been, by far, the most popular trigger molecule, cell-free riboswitches that respond to biotin,^77^ tetramethylrhodamine (TMR),^46,78,96^ flavin mononcleotide (FMN),^88,89,97^ cGMP,^36,87^ TPP,^39,42^ fluoride,^48^ histamine,^45^ dopamine,^46^ thyroxine,^46^ sulphorhodamine B,^88^ Hoechst dye 33258,^76^ tobramycin,^76^ tetracycline,^88,97^ and pentadeoxyribonucleotides^114^ have been designed (Tables 1 and 2). With the exception of histamine, however, these cell-free riboswitches have been constructed using known aptamers from natural riboswitches or discovered through SELEX intended for other applications. Additional aptamer–ligand combinations adapted to cell-free riboswitches will lead to better understanding of what makes an aptamer–ligand pair more suitable (or not) for cell-free riboswitches.

Finally, more efficient strategies for designing cell-free riboswitches are highly desirable. While many bacterial riboswitches have been shown to function similarly in prokaryotic cell-free systems, optimization of riboswitches in bacteria is not always possible because many desirable ligands for cell-free riboswitches may be toxic to the cells, impermeable through the cell wall or membrane, or unstable inside living cells. Most cell-free riboswitches not derived from bacterial analogs have been designed by trial-and-error or computational modeling, which can be laborious or unreliable. Since most successful design strategies for synthetic riboswitches in living cells involve some form of high-throughput screening or selection, a similar strategy for cell-free riboswitches should greatly facilitate the design process and improve performance. Availability of cell-free riboswitches that can interface cell-free systems with diverse chemical signals should accelerate development of innovative cell-free systems with complex functions and practical applications.

## Conflicts of interest

There are no conflicts to declare.

## Supplementary Material
